# Periodic Co/Nb pseudo spin valve for cryogenic memory

**DOI:** 10.3762/bjnano.10.83

**Published:** 2019-04-09

**Authors:** Nikolay Klenov, Yury Khaydukov, Sergey Bakurskiy, Roman Morari, Igor Soloviev, Vladimir Boian, Thomas Keller, Mikhail Kupriyanov, Anatoli Sidorenko, Bernhard Keimer

**Affiliations:** 1 Skobeltsyn Institute of Nuclear Physics, Moscow State University, Moscow 119991, Russia; 2 Moscow Institute of Physics and Technology, Dolgoprudny, Moscow Region, 141700, Russia; 3 All-Russian Research Institute of Automatics n.a. N.L. Dukhov (VNIIA), 127055, Moscow, Russia; 4 Max-Planck-Institut für Festkörperforschung, Heisenbergstraße 1, D-70569 Stuttgart, Germany; 5 Max Planck Society Outstation at the Heinz Maier-Leibnitz Zentrum (MLZ), D-85748 Garching, Germany; 6 Institute of Electronic Engineering and Nanotechnologies ASM, MD2028 Kishinev, Moldova; 7 Solid State Physics Department, KFU, 420008 Kazan, Russia

**Keywords:** cryogenic computing, neutron scattering, spin valve, superconducting spintronics

## Abstract

We present a study of magnetic structures with controllable effective exchange energy for Josephson switches and memory applications. As a basis for a weak link we propose to use a periodic structure composed of ferromagnetic (F) layers spaced by thin superconductors (s). Our calculations based on the Usadel equations show that switching from parallel (P) to antiparallel (AP) alignment of neighboring F layers can lead to a significant enhancement of the critical current through the junction. To control the magnetic alignment we propose to use a periodic system whose unit cell is a pseudo spin valve of structure F_1_/s/F_2_/s where F_1_ and F_2_ are two magnetic layers having different coercive fields. In order to check the feasibility of controllable switching between AP and P states through the whole periodic structure, we prepared a superlattice [Co(1.5 nm)/Nb(8 nm)/Co(2.5 nm)/Nb(8 nm)]_6_ between two superconducting layers of Nb(25 nm). Neutron scattering and magnetometry data showed that parallel and antiparallel alignment can be controlled with a magnetic field of only several tens of Oersted.

## Findings

Superconductor digital devices have attracted growing attention due to their unique energy efficiency and performance [1], and also due to compatibility with a number of quantum and neuromorphic computers under development [2–4]. However the lack of cryogenic memory elements (including synapses) with sufficiently fast switching between stable states and sufficiently small energy dissipation is still the main obstacle in the field. The utilization of the competition and coexistence of superconducting (S) and ferromagnetic (F) correlations could provide an increase in the performance and degree of integration for cryogenic memory storage devices and synaptic elements [1,5–16]. These ideas can be implemented through a Josephson contact with two stable states: a high value of the critical current, *I_C_*, corresponds to the ”open” state and a low value to the ”closed” state. Such a device can be assembled if the weak link is a composite F/N/F trilayer (N is a normal metal) whose magnetic state can be switched between parallel and antiparallel directions of the magnetization vectors of the F layers [7].

**Figure 1:**
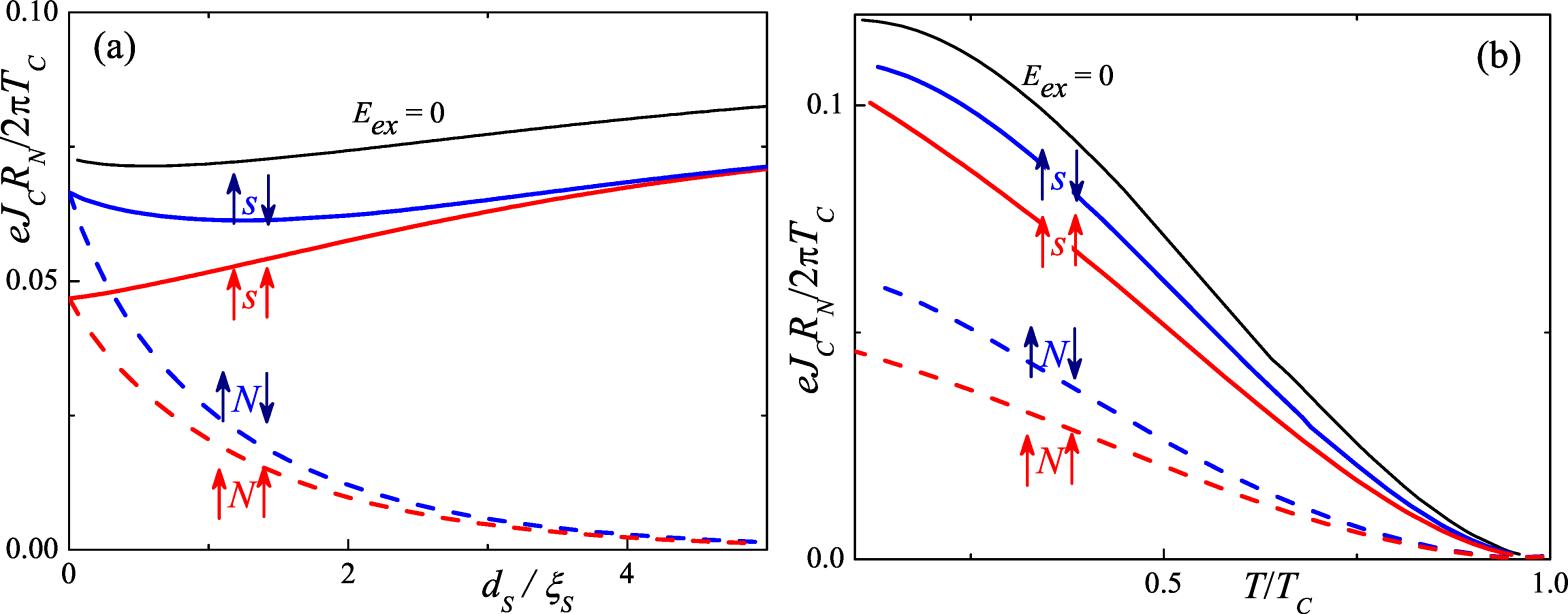
The normalized critical current of the S/F/s/F/S (solid lines) and S/F/N/F/S (dashed lines) structures as a function of the thickness of the spacing layer (a) and the dimensionless magnitude of the temperature (b). The red lines correspond to the case when the exchange energies in both layers of the ferromagnet are equal in magnitude, and the magnetization vectors lying in the plane of the magnetic layers are parallel (P). The blue lines are for the case when the exchange energies, *E_ex_*, in both F-layers are equal, and the described magnetization vectors are antiparallel (AP). The black curves correspond to the *E_ex_* = 0 case.

The use of a thin superconducting layer (s) as a spacer instead of an N layer may lead to enhancement of the spin-valve effect due to the proximity of the thick superconductor banks and the thin superconductor spacers (see, e.g., [8]). To check this hypothesis we calculated the critical current of S/F/s/F/S and S/F/N/F/S Josephson junctions (Figure 1). The calculations were performed in the framework of the Usadel equation [17]

[1]
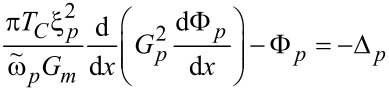


with Kupriyanov–Lukichev conditions [18]

[2]
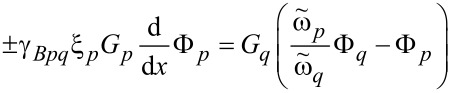


in an iterative manner with respect to the pair potential Δ to ensure fulfilment of the self-consistency equation

[3]
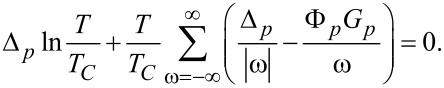


Here *p* and *q* are the indices of corresponding layers, 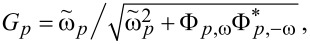 
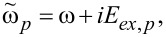
ω= π*T*(2*n* + 1) are the Matsubara frequencies, Δ*_p_* is the pair potential (which is absent in the F and N layers), *E_ex,p_*, is the exchange energy (*E_ex,p_* = 0 in nonferromagnetic materials), *T_C_* is the critical temperature of the superconductor, ξ*_p_* = (*D_p_*/2π*T_C_*)^1^*^/^*^2^ is the coherence length, *D_p_* is the diffusion coefficient, *G_p_* and Φ*_p_* are the normal and anomalous Green’s functions, respectively, 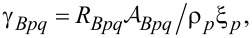 is the suppression parameter, *R_Bpq_* and 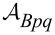 are the resistance and area of the corresponding interface. The plus sign in Equation 2 means that the *p*th material is located at the side *x_i_* − 0 from the interface position *x_i_*, and the minus sign corresponds to the case that the *p*th material is at *x_i_* + 0, while the *x* axis is oriented perpendicular to the interfaces. Finally, the boundary conditions on the surfaces of the outer electrodes are 
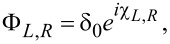
 where δ_0_ is the bulk value of the pair potential at a certain temperature and χ*_L,R_* are the phases at the left and right ends of the structure, which generate the phase difference φ = χ*_R_* − χ*_L_* along the junction.

Figure 1a and Figure 1b show the dependence of *I_C_* on the reduced thickness of a spacer, *d_s_*/ξ*_S_*, and temperature, *T*/*T_C_* for parallel (P) and antiparallel (AP) orientations of the F films’ magnetization vector, **M**. It should be noted that unlike in references [19–20] our approach obtains a solution for the Green’s functions, which already corresponds to the state with minimal free energy and automatically determines which of the states, either 0 or π , is energetically favorable on each junction. At the same time, in the systems with multiple junctions connected in series, there are multiple stable solutions differing by 2π*n* in the phase of the outer S electrodes. To avoid any errors we calculate the critical current dependence in an iterative manner over the phase difference, initially solving the problem at φ = 0 and then continuously increasing the phase, using the results of the previous step as the initial function for the solution of Equation 1, Equation 2 and Equation 3.

As it follows from Figure 1 the existence of intrinsic superconductivity of the spacer significantly increases *I_C_* of the S/F/s/F/S compared to the S/F/N/F/S junction. The effect can be essentially enhanced in S/[F/s]*_n_*/F/S Josephson devices with the superlattice in the weak link region. The use of a multilayer structure has several advantages. Thanks to the collective effect of maintaining the superconducting state in the spacers, it is possible to use thinner layers. The thinning of the layers should be accompanied by a decrease in the effective exchange energy due to its renormalization [21–22]. Moreover, for the AP orientation of the magnetization vectors of the F layers, an additional mechanism arises for the renormalization of the effective exchange energy, which leads to its further decrease [23–25].

**Figure 2:**
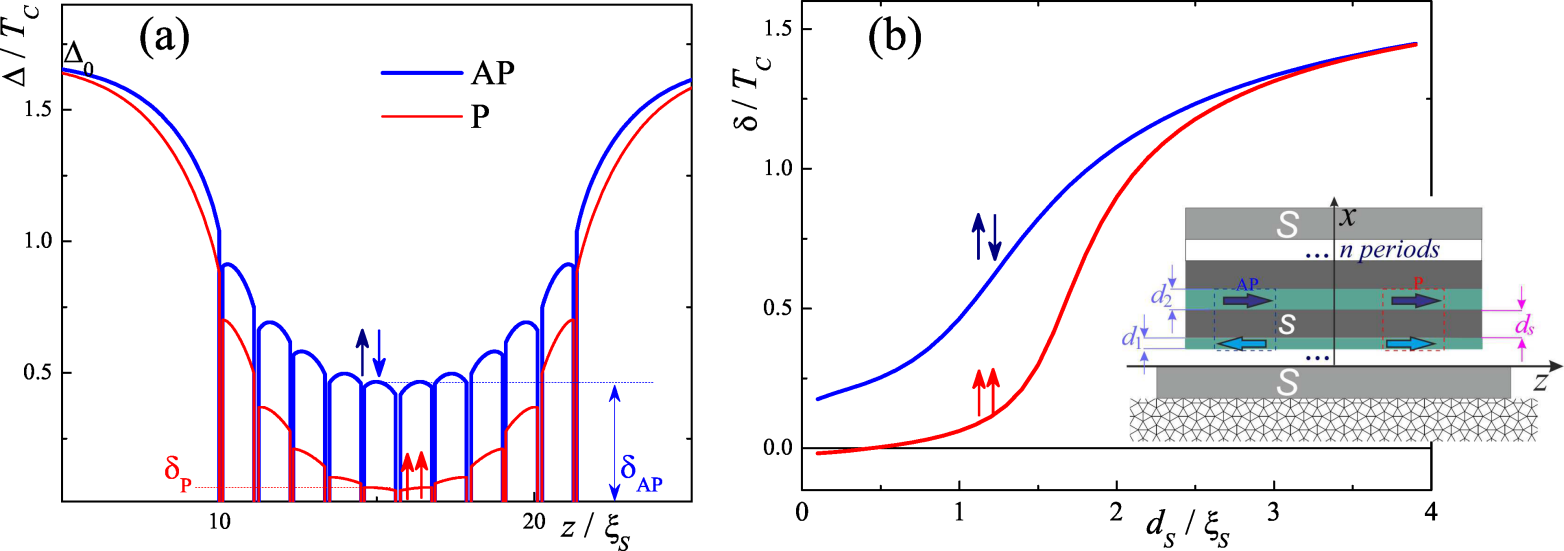
(a) The depth profile of the superconducting pair potential amplitude of the S/[F_1_/s/F_2_/s]_5_/F_1_/S structure in the P and AP cases. (b) The amplitudes of the superconducting pair potential in the middle of the weak link for the same situations. Inset – schematic representation of the considered stack structure.

To confirm these statements, we have generalized the S/[F/N]*_n_* model [25] to the case of the existence of intrinsic superconductivity in its non-ferromagnetic parts. To make the model more realistic we consider a case of a periodic pseudo-spin-valve structure, where two neighboring F layers have slightly different thicknesses *d*_1_ and *d*_2_ (see inset in Figure 2b). The difference in the thicknesses of the F_1_ and F_2_ layers provides a difference in their coercive fields *H*_c1_ ≠ *H*_c2_ which allows one to organize an AP state in the range of magnetic fields, *H*, max(*H*_c1_,*H*_c2_) > *H* > min(*H*_c1_,*H*_c2_) after saturation of the layer magnetization in the negative direction. Thus the use of the pseudo-spin-valve concept allows us to organize AP alignment without exchange or magnetostatic coupling of neighboring F layers. Figure 2a shows the spatial distribution of the pair potential amplitudes in the S/[F_1_/s/F_2_/s ]*_n_*/F_1_/S structure for the P and AP alignments. The calculations were performed for the same set of parameters as in Figure 1. From Figure 2a it follows that the considered structure is a series connection of s/F_1_/s and s/F_2_/s Josephson junctions with the weakest link located in the middle of the structure. Figure 2b shows the amplitudes of the pair potential, δ_P_, and δ_AP_, (see the definition of δ_P_ and δ_AP_ in Figure 2a) in the middle of the weak link as a function of the s layers’ thickness. One can see that the amplitudes for AP and P configurations are significantly different for *d_s_* *~* ξ*_S_*. As soon as *I_C_* is proportional to the product of the pair potential amplitude of the s banks, one may estimate that the ratio of *I_C_* for AP orientation and P orientations is of the order of (δ_AP_/δ_P_)^2^ ≈ 25. From Figure 2b it follows that this enhancement depends on the ratio *d_s_*/ξ*_S_* and is maximal in the vicinity of *d_s_* = ξ*_S_*.

Realization of the proposed S/[F_1_/s/F_2_/s]*_n_*/F_1_/S Josephson devices requires the development of a technology for manufacturing of multilayer structures that satisfy the following conditions: (a) presence of superconductivity in the s layers with *T_C_* 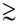 4.2 K, (b) in plane orientation of the magnetization vector in the F films, and (c) ability for coherent switching between P and AP configurations through the whole stack. The goal of this paper is to demonstrate that the requirements can be met when using a combination of Nb and Co as materials for the superlattice. To do this we fabricated a Nb(25 nm)/[Co(1.5 nm)/Nb(8 nm)/Co(2.5 nm)/Nb(8 nm)]_6_/Co(1.5 nm)/Nb(25 nm) structure. We took niobium as a superconducting material since it has the highest *T_C_* = 9.25 K among all elemental superconductors and forms stable structures with cobalt [19,26–30]. The thickness of the Nb-spacer was chosen to be close to ξ*_S_* ≈ 6–10 nm, the value found in our prior studies [31–32]. The thickness of the Co layers were in the range of ξ*_F_* ≈ 1 nm [19], which is enough to form a homogeneous and magnetic layer [26].

The sample was prepared using a Leybold Z-400 magnetron machine at room temperature on an R-plane-oriented sapphire (Al_2_O_3_) substrate. Before the deposition the substrates were etched by an argon ion beam inside the chamber. The targets Nb(99.99%) and Co(99.99%) were presputtered to remove metallic oxides and contamination absorbed on the surfaces. Additionally, immediately before deposition of the next layer we presputtered the corresponding target for 40–50 seconds for stabilization of the film growth rate. The deposition was performed in a pure argon atmosphere (99.999% purity) at a working pressure of 8 × 10^−3^ mbar. The thickness of the films was controlled by the time of deposition of the material on the substrate. For high repeatability of the thicknesses of thin Nb films, an electrical motor was used to move the target above the substrate at an equal speed so that the thickness of the niobium layer remains the same for each of the periods of the structure. The growth film rate is 1 nm/s and 0.1 nm/s for Nb and Co, respectively. After the deposition the structure was capped by a silicon layer.

**Figure 3:**
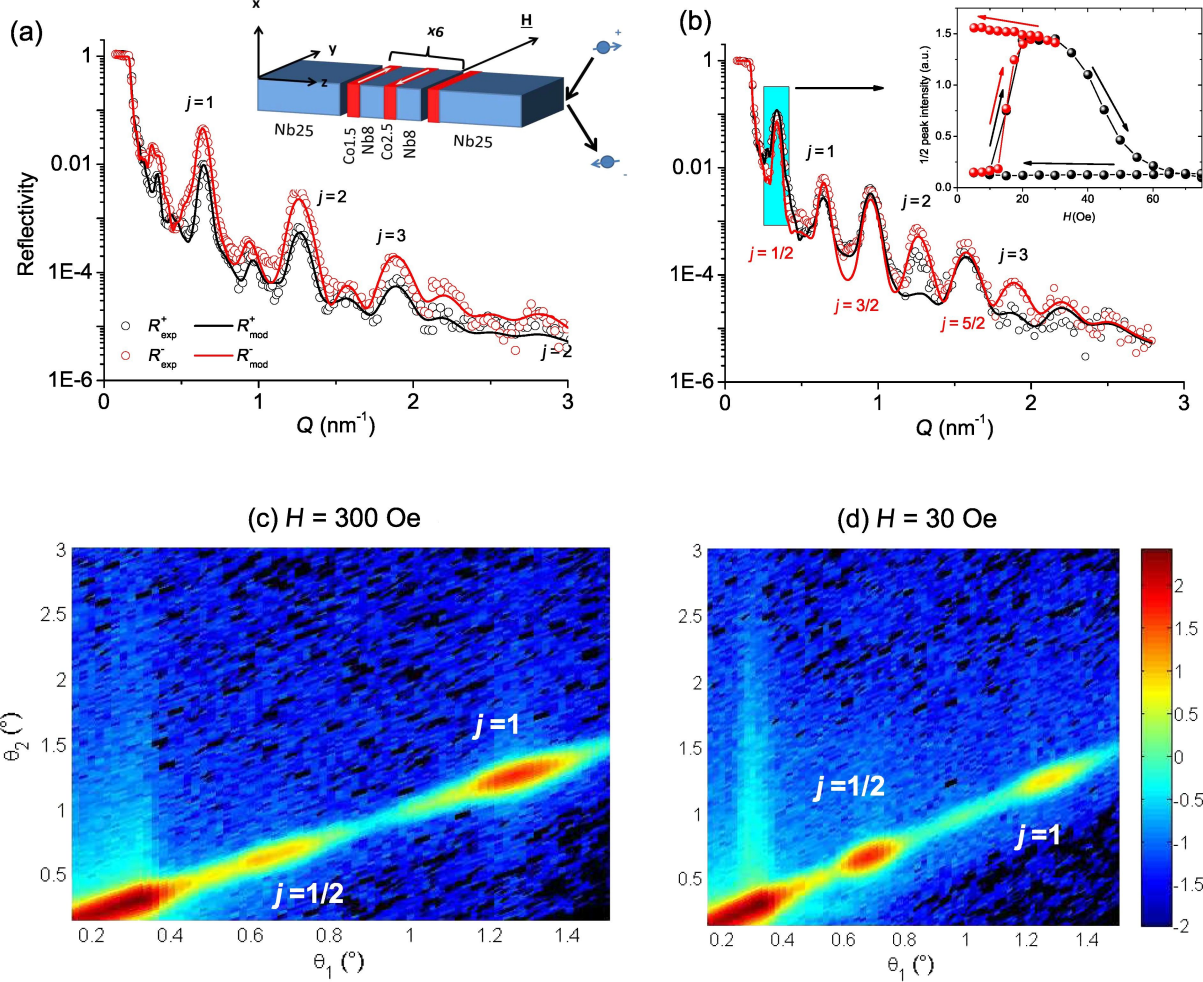
Experimental (dots) specular neutron reflectivity measured at *T* = 13 K in magnetic fields *H* = 300 Oe (a) and *H* = 30 Oe (b). Solid lines show the model curves for the magnetization depth profiles depicted in Figure 4a. The inset in (a) shows the sketch of the sample and experimental setup. The numbers above show the corresponding order of Bragg reflection from the effective [Co(2 nm)/Nb(8 nm)] × 12 periodic structure. The inset in (b) shows the field dependence of the *j* = 1/2 peak shown by the blue rectangle in (b). The logarithm of intensity of spin-down scattered neutrons measured at *H* = 300 Oe and *H* = 30 Oe is shown in (c) and (d).

In order to characterize the structural and magnetic ordering of the Co/Nb superlattice we performed polarized neutron reflectometry (PNR) experiments. The measurements were conducted at the neutron reflectometer NREX at the research reactor FRM-2 (Munich, Germany). The neutron reflectivities were taken with a monochromatic polarized neutron beam of wavelength λ = 0.43 nm at a temperature *T* = 13 K with the magnetic field applied in-plane to the structure and normal to the scattering plane (see inset in Figure 3a). No spin analysis of the scattered beam was performed in this experiment. Figure 3a and Figure 3b shows reflectivities measured at *H* = 300 Oe and in magnetic field *H* = 30 Oe after magnetization of the sample in the negative direction. The curves in the saturated state are characterized by Bragg peaks positioned at *Q_i_* ≈ 2π × *i*/(*d*_1_ + *d*_2_ + 2*d_s_*) (*i* = 1–7). However, one can see that the odd-integer peaks are of quite small intensity compared to the even-integer peaks due to the small difference between the Co(1.5 nm)/Nb(8 nm) and Co(2.5 nm)/Nb(8 nm) bilayers within the unit cell. In this regard we can effectively consider our periodic structure as [Co(2 nm)/Nb(8 nm)]_12_ and index the Bragg peaks using the notation *Q_j_* ≈ 2π × *j*/10 nm (*j* = 1,2,3). The reflectivity pattern at *H* = 30 Oe strongly differs from the saturated state. First of all we can see the growth of non-integer peaks *j*/2 which directly evidence the doubling of the magnetic period at this field [33–37]. The small difference of the *R*^+^ and *R*^−^ peaks indicates compensation of the magnetic moments of neighboring Co layers, e.g., antiparallel alignment. The inset in Figure 3b shows the field evolution of the *j* = 1/2 peak. One can see that the AP alignment exists in the range of magnetic fields *H* = 10–30 Oe if the sample is firstly magnetized in the negative direction. Moreover once the AP state is created the field can be returned to zero and the alignment will be preserved. The P-alignment can also be organized at zero field if the sample is saturated before in a positive field.

**Figure 4:**
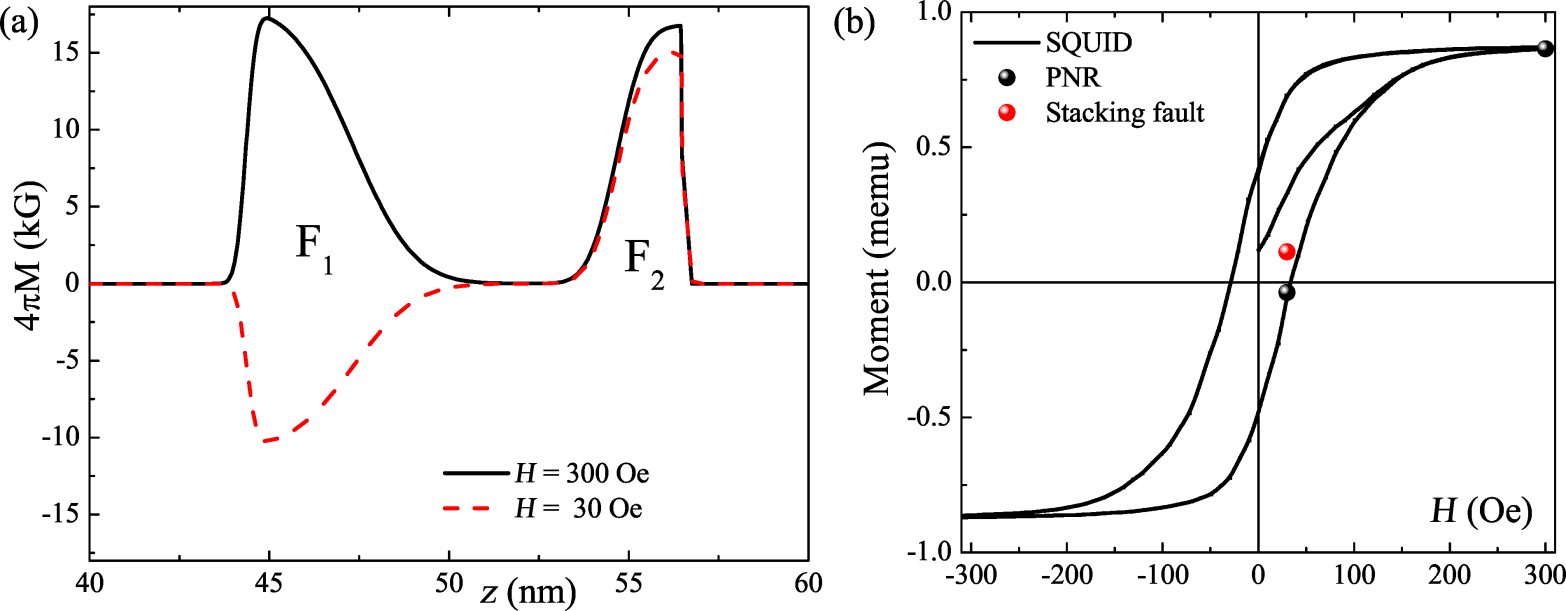
(a) Magnetic depth profiles of one unit cell for the P (black) and AP (red) alignment. Corresponding neutron curves are shown by solid lines in Figure 3a. (b) Hysteresis loop measured by SQUID magnetometry (solid line). The black dot indicates the magnetic moment of the sample which is obtained by the integration of the depth profiles depicted in (a). The red dot shows the magnetic moment at *H* = 30 Oe expected if one P segment were present in the AP aligned structure.

In a periodic pseudo-spin-valve structure one cannot exclude noncoherent switching of the F layers. Such a stacking fault in the antiferromagnetically aligned system may lead to the suppression or even destruction of the spin-valve effect. In order to check the presence of stacking faults in our system we performed a comprehensive analysis of PNR and superconducting quantum interference device (SQUID) magnetometry data (Figure 4). To fit the experimental data we considered a simple model of a Co(1.5 nm)/Nb(8 nm)/Co(2.5 nm)/Nb(8 nm) quadrolayer repeated six times. First we fitted data in the saturated state varying both nuclear and magnetic depth profiles. Then the data at *H* = 30 Oe were fitted varying only the magnetic depth profile. Figure 4a shows the resulting magnetization depth profiles for the model curves 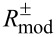 depicted by solid lines in Figure 3a. One can see that despite the simplicity of the model it describes the experimental curves reasonably well. Moreover the derived magnetic depth profiles for both P and AP states agree well with the SQUID magnetometry data (Figure 4b). If we consider the presence of at least one ferromagnetically aligned segment in the AP aligned lattice, this will lead to a substantial increase of the total magnetic moment (see the red dot in Figure 4b) which is in strong disagreement with the SQUID data. Thus we rule out the presence of stacking faults in our sample.

Thus in this work we considered the possibility to control the superconducting properties of Josephson junctions by switching between parallel and antiparallel alignment in a periodic F/s weak link. We experimentally showed that such a switching is feasible using the concept of a periodic pseudo spin valve. We note that such a design will allow us in the future to study the possible influence of superconductivity on the magnetic configuration via electromagnetic [38–39] or exchange [40–41] mechanisms.

In conclusion, we have proposed a memory element based on a Josephson junction with a weak link composed of a periodic S/F structure that that can be switched between AP and P states. In the framework of the Usadel equations we showed that the critical current across the junction significantly depends on the magnetic state of the periodic structure. In order to switch between AP and P states we propose to use a periodically repeated quadrolayer F_1_/s/F_2_/s where the magnetic layers F_1_ and F_2_ have slightly different coercive fields. In order to experimentally investigate the switching processes between P and AP states we sandwiched the periodic structure [Co(1.5 nm)/Nb(8 nm)/Co(2.5 nm)/Nb(8 nm)] × 6/ Co(1.5 nm) between two Nb(25 nm) electrodes. Using neutron reflectometry we demonstrated that an AP state can be created and erased by applying a field of 30 Oe.
